# Characterizing the Discussion of Antibiotics in the Twittersphere: What is the Bigger Picture?

**DOI:** 10.2196/jmir.4220

**Published:** 2015-06-19

**Authors:** Rachel Lynn Kendra, Suman Karki, Jesse Lee Eickholt, Lisa Gandy

**Affiliations:** ^1^ Department of Computer Science Central Michigan University Mount Pleasant, MI United States

**Keywords:** Twitter messaging, social media, Internet, Web mining, semi-supervised learning, neural network

## Abstract

**Background:**

User content posted through Twitter has been used for biosurveillance, to characterize public perception of health-related topics, and as a means of distributing information to the general public. Most of the existing work surrounding Twitter and health care has shown Twitter to be an effective medium for these problems but more could be done to provide finer and more efficient access to all pertinent data. Given the diversity of user-generated content, small samples or summary presentations of the data arguably omit a large part of the virtual discussion taking place in the Twittersphere. Still, managing, processing, and querying large amounts of Twitter data is not a trivial task. This work describes tools and techniques capable of handling larger sets of Twitter data and demonstrates their use with the issue of antibiotics.

**Objective:**

This work has two principle objectives: (1) to provide an open-source means to efficiently explore all collected tweets and query health-related topics on Twitter, specifically, questions such as what users are saying and how messages are spread, and (2) to characterize the larger discourse taking place on Twitter with respect to antibiotics.

**Methods:**

Open-source software suites Hadoop, Flume, and Hive were used to collect and query a large number of Twitter posts. To classify tweets by topic, a deep network classifier was trained using a limited number of manually classified tweets. The particular machine learning approach used also allowed the use of a large number of unclassified tweets to increase performance.

**Results:**

Query-based analysis of the collected tweets revealed that a large number of users contributed to the online discussion and that a frequent topic mentioned was resistance. A number of prominent events related to antibiotics led to a number of spikes in activity but these were short in duration. The category-based classifier developed was able to correctly classify 70% of manually labeled tweets (using a 10-fold cross validation procedure and 9 classes). The classifier also performed well when evaluated on a per category basis.

**Conclusions:**

Using existing tools such as Hive, Flume, Hadoop, and machine learning techniques, it is possible to construct tools and workflows to collect and query large amounts of Twitter data to characterize the larger discussion taking place on Twitter with respect to a particular health-related topic. Furthermore, using newer machine learning techniques and a limited number of manually labeled tweets, an entire body of collected tweets can be classified to indicate what topics are driving the virtual, online discussion. The resulting classifier can also be used to efficiently explore collected tweets by category and search for messages of interest or exemplary content.

##  Introduction

The development and proliferation of social media and social media networks have transformed how information is generated and shared. Participants in social media are actively engaged and are both consumers and producers of information. Toffler et al label these users “prosumers” [1]. The Pew Research Center reported in January 2014 that 74% of online adults use social networking sites. They also report that 46% of adult Internet users post original photos or videos online that they have created [2]. This content posted by users is of great utility, and several studies have demonstrated how social media and networks can be a valuable source of data. Furthermore, the nature and ubiquity of social media and how it is so interwoven in daily life means that the topics covered span the spectrum. For example, trending topics on Twitter in December 2014 were the Siege in Sydney, Australia, the antics of Rubius Gunderson, a popular prankster on YouTube, and news regarding Ross Barkley, who is a popular soccer player from the English National Team.

Twitter is a social media platform through which users post status updates called tweets [3]. A tweet can contain up to 140 characters and can be public (ie, any visitor can access and view the tweet) or protected (ie, only approved visitors can view the tweet). As of October 2012, approximately 88% of Twitter accounts were public [4]. Users can “follow” other users and thus be apprised of any new tweets that the followed users post, and a user may be followed by any number of other users. Users can also repost tweets pushed out by users they follow. This action is known as retweeting and can lead to a message “going viral”, a phenomenon that quickly spreads the reach of a message [5]. Another way that users can increase the reach of their message is through hashtags (a continuous string of characters that begin with a #). Hashtags are often used to hint at the content of a tweet and provide an additional means to tie related tweets together.

Twitter users tweet about a variety of subjects including health-related topics. Such tweets may share information about health articles or describe personal health issues. A number of approaches have been developed to extract useful health-related information from the tweets, evaluate the effectiveness of Twitter for disseminating health-related information, and determine public sentiment towards health-related topics [6-12]. Love et al did an extensive study on 6827 tweets related to vaccinations to determine the source of the information and the medical claims made [10]. They found that no particular source or medical claim dominated the content shared regarding vaccinations and that 87% of user posts were positive or neutral. Scanfeld et al manually inspected and determined a number of topic-based categories present in tweets related to antibiotics. In doing, so several examples of misunderstanding or misuse were detected based on keyword combinations (eg, “antibiotics” and “flu”) [11]. Furthermore, Vance et al explored the concept of using social media to disseminate public health information to young adults [12]. Advantages to such an approach included rapid communication and low cost. The drawbacks cited were opinions often being represented as facts, the use of blind authorship, and a lack of citations.

An additional use of Twitter data is biosurveillance [13,14]. Self-reported behaviors can be monitored and used to detect epidemics or break-outs in real time through crowd sourcing. For example, Google Flu Trends tracks the influenza rates by tracking user queries on a daily basis, and their system is usually 7-10 days faster than the Centers for Disease Control and Prevention [15]. Lampos and Cristianini found a correlation between tweets about the flu and historical data from the same time period, further strengthening the claims that Twitter can be used for biosurveillance [16].

In this work, we focused on accessing and mining topics in the Twittersphere with open source tools. In doing so, we had two objectives in mind. First, we wanted to characterize the exchange currently taking place on Twitter with respect to a particular topic. Of particular interest was examining what was dominating the virtual discussion and if it was being dominated by a small set of users. In answering these questions, we wanted to leverage as much of the virtual discussion as possible. As a result, the second objective of this work was to develop tools and workflows to access the larger Twittersphere. To this end, we developed a classifier that can be used to identify and draw out tweets pertaining to several categories. The classifier was trained using a semisupervised approach that allows it to make use of all of the collected tweets during the learning process. We also describe a number of tools and techniques for handling the larger amounts of data. In particular, Hadoop and Hive were used to query and characterize the large number of tweets that were collected. The resulting pipeline could be used as a tool for infodemiology and infoveillance, providing a means of ferreting out sources of information or specific types of messages shared through Twitter. Given the ease at which Hadoop scales and the availability of cloud computing platforms, this approach could easily be applied to much larger datasets and other topics.

As a case study, the pipeline and tools developed in this work were applied to the topic of antibiotics and antibiotic resistance. By querying 591,091 tweets with the workflow, the current discussion surrounding antibiotics could be characterized and types of misuse examined. This revealed that there were a large number of unique participants in the online discussion and that the discussion was not being dominated by a set of users but rather by a large number of users who were sharing national news stories. The topic of antibiotics was chosen given the potential economic costs associated antibiotic resistance in bacteria [17,18] and its dominance in the media (eg, Longitude Prize). Additionally, with the number of existing studies on Twitter and antibiotics, this topic provided a context in which our tools, methodology, and findings could be compared and contrasted.

##  Methods

### Data Collection

To collect tweets related to antibiotics, the Twitter Application Programming Interface (API) was used in conjunction with a list of 89 antibiotic-related terms and Apache Flume [19]. These terms included expected keywords such as antibiotic(s) as well as common abbreviations (eg, abx), names of specific antibiotics (eg, amoxicillin, penicillin), and common misspellings (eg, antiboitic). Collection of the tweets began on May 27, 2014, and ended September 11, 2014. These dates were selected to provide a minimum collection period of 3 months. Additionally, the number of tweets collected was periodically checked and an aim of collecting over 500,000 tweets was also taken into account when determining the date to stop collection. Note that the content of the tweets was not examined during the collection period and consequently did not affect the collection dates. Over this period, 591,091 tweets were collected and then subjected to a post-collection filtering process. This was needed to remove a large number of unrelated tweets that were received due to the keyword “abx”, which also referred to an active stock symbol.

### Data Analysis and Associated Tools

Hadoop [20] and Apache Hive [21] were used to handle the large number of tweets. Hive is a software tool that allows for query-based processing of large amounts of data through Hadoop and accessible through HiveQL, a language similar to SQL (structured query language). All of the tweets collected were saved on a local, distributed file system in their native JSON format as provided through the Twitter API. From the tweets, a table was constructed in Hive with the columns of the table based on the properties of a tweet, such as the tweet text, user information, location information, and so forth. From there, queries were run pulling selected data from the table using HiveQL. With Hive, the queries were automatically converted to run as MapReduce tasks within Hadoop. With Hadoop and the distributed file system, it was possible to process the roughly 600,000 tweets collected in a timely matter (ie, seconds to minutes depending on the complexity of the query) on a modest Hadoop cluster (eg, 4 computing nodes containing 64 computing cores and 132 GB of RAM).

**Figure 1:**
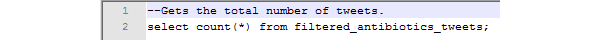
HiveQL query to count the total number of tweets.

**Figure 2:**
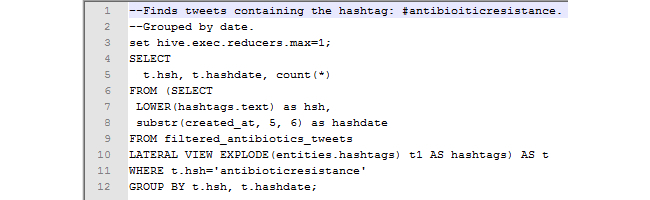
HiveQL query to determine the number of tweets containing "#antibioticresistance", sorted by date.

To illustrate the relative ease by which the data can be queried, two sample queries are provided. Figure 1 is an example of a simple HiveQL query. This query finds the number of total tweets within the table of filtered antibiotics tweets. Figure 2 is a more complex query and finds the number of tweets that contain the hashtag “#antibioticresistance”. It then sorts the tweets by day to get a count of the number of tweets that contained the hashtag on each day in our collection period. This query required a nested select statement, which, when converted as a MapReduce, requires two passes through the data. With Hive, large amounts of data can be efficiently queried by anyone familiar with an SQL style database.

### Manual Classification Procedure for Tweets

An explicit aim of this work was to develop a means to classify tweets and thus provide efficient and finer-grain access to the larger pool of tweet data. Manual classification is impractical as well as inefficient for processing and categorizing a large number of tweets. As a result, a deep network was trained and used to classify antibiotic-related tweet data collected from Twitter into 9 classes. The first step in developing a topic-based classifier was to manually label a portion of the tweets. This was done by randomly sampling 1000 tweets and then manually classifying them. The categories used were inspired by Scanfeld et al [11]. The manual classification task was undertaken by 3 individuals and done independently. Fleiss’ kappa was calculated to measure the agreement between the 3 manual coders and the value was .47. After the individual classification, a tweet was added to the labeled set if at least two of manual classifications agreed. This process led to a labeled dataset of 416 tweets with an uneven distribution over the classes. Table 1 lists the categories considered and an example of each from the labeled dataset. An additional evaluation set was also constructed by randomly sampling 300 tweets and performing the same manual classification. This resulted in a set of 246 tweets and the distribution of tweets by category was similar to that of the principle training set.

**Table 1:** Categories considered for antibiotic-related tweets, quantities of each in the labeled dataset, and examples.

Category	Count	Example
Advertisement	21	#celebrex: Amoxicillian Antibiotic: Generic Amoxil – Antidepressant Celebrex OMITTED_URL
Advice/Information	88	Big pharma not interested in risk and low return of developing new antibiotics
Animals	28	RT @USERID: 80% of all antibiotics in the US are used on farm animals. OMITTED_URLS
General use	38	Bronchitis has got the best of me, Dr’s orders to stay home rest and lots of liquids with the antibiotics.
Other	72	How to know you’re in the medical field: Seeing the work ‘piper’ and thinking of piperacillin #nurse
Resistance	132	Antibiotic-resistant superbugs threaten return to ‘dark ages’.
Side effects	16	Being put on these antibiotics did way more harm than good.. My stomach has never hurt so bad. Never again. ?
Wanting/Needing	15	Cant wait to get some antibiotics tomorrow from the doctors, can finally get back to normal!!
Misuse	7	Idk if im allowed to mix this Vicodin and this antibiotic. I forgot to ask my dr… oh well.
Total	416	

### Construction of a Topic-Based Classifier

To classify all of the tweets, an in-house software package was used to train a deep network. In recent years, deep neural networks have become one of the most popular and powerful techniques in machine learning for classification tasks. Compared with other techniques such as support vector machines and neural networks, deep neural networks typically perform better [22,23] due to their ability to learn high-level abstractions and correlations present in labeled and unlabeled datasets (ie, labeled datasets are those in which each example has a known category/class). In particular, DNs are able to learn patterns present in unlabeled datasets through a layer-by-layer initialization task. This ability to use unlabeled data is particularly advantageous in this setting given the large amount of unlabeled tweets. To date, deep networks and deep learning architectures have been successfully applied in several areas such as speech recognition [24], image classification [23], protein structure prediction [25], and natural language processing [26]. In the health and medical fields, deep networks and deep learning are also gaining traction with applications in computer-aided diagnosis (eg, Alzheimer’s Disease) [27], automatic segmentation of diagnostic images (eg, neurological structures in electromagnetic scans) [28,29], lymph node detection from computerized tomography scans [30], and clustering descriptions of adverse drug reactions [31].

In selecting features used to characterize a tweet, a bag-of-words approach was used along with a few global properties such as tweet length or the presence of a URL. Specifically, the text of each tweet was stemmed using a Snowball stemmer included in the Natural Language Toolkit [32] and the presence or absence of several common stems was encoded. To identify the stems used, the most common stems contained in each class (and in general) were determined using HiveQL. Up to the top 50 stems per class and the top 1000 stems overall were used to generate the bag-of-words. Additionally, 10 features were used to encode the length of the tweet (ie, one feature represented bins of 0-9, 10-19, 20-29, etc, respectively) and one binary feature was used to encode the presence of a uniform resource locator (URL) in the tweet. In total, the number of features was 1383.

The full training dataset contained 412 manually labeled examples and 150,000 randomly sampled tweets as unlabeled training examples. Again, a principle advantage of deep networks is the ability to use large amounts of unlabeled data in a semisupervised manner. The overall architecture of the deep network used consisted of 5 layers (ie, an input layer, 3 hidden layers, and an output layer of 11 nodes). The input layer consisted of 1383 features, and the three hidden nodes contained 700, 700, and 300 sigmoid nodes.

### Training, Evaluation, and Application of the Classifier

To train and evaluate the deep network model, stratified 10-fold cross validation or so called rotation estimation was used. The labeled dataset was split into 10 sets using stratified sampling techniques, and 9 folds were used for training and the other held out for evaluation. In the training phase, the model was first pre-initialized using the unlabeled data and a layer-by-layer training procedure making use of Restricted Boltzmann Machines [23]. After initialization, the model was fine-tuned using standard back propagation and the labeled data. This process was repeated 10 times in order to make predictions over all the labeled dataset (ie, a prediction for each labeled example was made using 9/10 of the labeled data, which excluded the examples being evaluated). For fine-tuning of the model, a batch size of 10 was used and the refinement took place over 200 epochs. To increase the robustness of the classifier and guard against over-fitting, a dropout procedure was used [33].

To apply a classifier to all collected tweets, a final model was created using all 10 labeled datasets and the aforementioned training procedure (ie, layer-by-layer initialization with 150,000 unlabeled data followed by fine-tuning). Features were generated for all 591,091 tweets and then run through the deep network classifier. The result was a score for each tweet and each category. The total sum for the scores of a tweet across categories was 1.0 (ie, the last layer in the deep network was a multinomial node with 9 classes). For each category, a higher score corresponded to a more confident prediction for that particular category. To classify all tweets, a tweet can be assigned to the highest scoring category. To search the full dataset for tweets pertaining to a particular category, a variable threshold can be chosen (the higher the threshold, the more confident the predictions for the recovered tweets) and all the text for all tweets meeting a particular threshold can be recovered.

## Results

### Characterizations of Collected Tweets via Hive Queries

**Figure 3:**
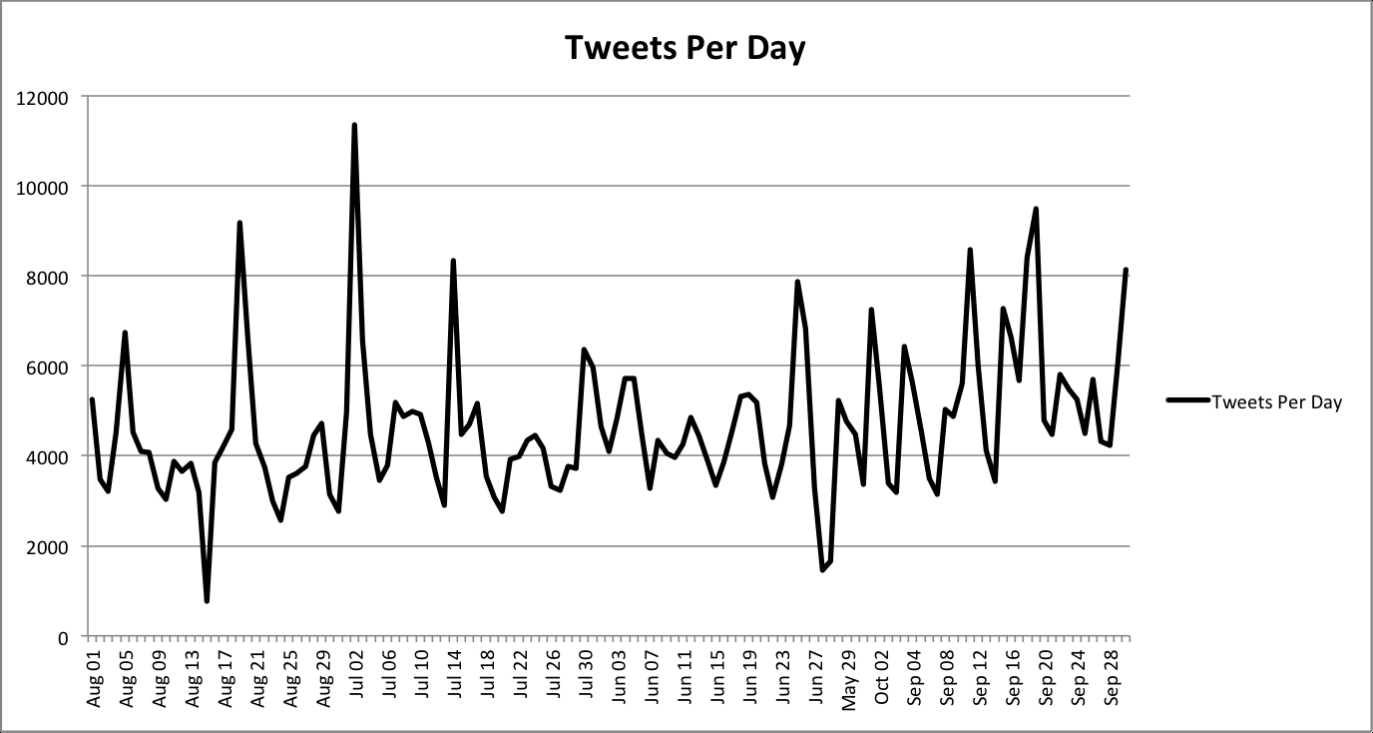
Number of antibiotic-related tweets collected per day.

To begin to characterize the exchange currently taking place on Twitter with respect to antibiotics, a number of HiveQL queries were performed. First, all collected tweets were collated and counted by date posted to determine a baseline for tweet activity. There was an average of 4654.3 tweets per day. The day with the most activity had 11,365 tweets, and activity usually ranged between 3055 and 6253 (ie, mean +/- standard deviation). Figure 3 illustrates the number of tweets per day during the collection period. There were 8 days with an unusually high number of antibiotic-related tweets (ie, the *Z* score for the number of tweets >2.0). For each of these days, the tweets posted were collected, sorted, and inspected to determine what may have driven the spike in activity. A summary of these dates is contained in Table 2. By examining the most occurring words and retweeted messages by day, it was possible to describe the general cause for the increased activity. On July 2, the day with the most activity, many tweets focused on a speech given by the Prime Minister of the United Kingdom. The second and fifth most active days, September 19 and 18, had tweets related to actions made by US President Obama to battle against antibiotic resistance. On August 19, activity was inflated by an advertisement that was retweeted over 2600 times. In general, it was a news story that led to the increased amounts of tweeting but advertisements did contribute to higher than normal activity on more than one occasion. Note that the general topic for a day was determined by the contents of the tweets on these days of high activity and not by determining a specific source (eg, a particular URL or online news outlet).

**Table 2:** Dates with unusually high tweet count along with rationale for activity.

Date	Tweet count	General topic	Sample of most frequent words	Sample tweet
July 2	11365	Comments made by British PM regarding resistance	resistance (3411), pm (1610), cameron (1264), warns (1330), dark (965), ages (916)	Antibiotic resistance: Cameron warns of medical ‘dark ages’ URL #health #antibioticresistance #evolution
Sept 19	9489	Executive action from US president on antibiotics	resistance (2342), obama (1394), bacteria (1112), order(946), plan(780), president (747)	RT @PublicHealth: New national strategy, presidential executive order take aim at antibiotic resistance: URL
Aug 19	9188	Advertisement	antibiotics (4718), celphalexin (2657)	RT @...: Buy cephalexin URL
Sept 11	8589	News about a bee-based alternative to antibiotics	antibiotics (5093), bacteria (830), honey (713), alternative (603)	RT @Independent: Bacteria found in honeybee stomachs could be used as alternative to antibiotics
Sept 18	8412	Executive action from US president on antibiotics	resistance (2347), combat (1294), Obama (1070), bacteria (940), strategy (1050)	New Executive Actions to Combat Antibiotic Resistance and Protect Public Health: Today, the Obama admin … URL #obama
July 14	8340	Advertisements	doxycycline (2229), health (1459), treating (1360), bronchitis (1358),	RT @...: Treating Acute Bronchitis and the Use of Antibiotics URL
Sept 30	8147	Report that antibiotics increases risk for obesity	antibiotics (4394), obesity (2827), childhood (1356), study (946)	Antibiotics in infancy may be linked to childhood obesity: study URL
June 25	7871	News breaks that the focus of the Longitude Prize will be antibiotic resistance	antibiotic (2242), resistance (1619), prize (1615), longitudeprize (1089)	RT @longitude_prize: The votes have been counted and the results are in – the challenge of Longitude Prize 2014 will be …antibiotics! #longi…

**Figure 4:**
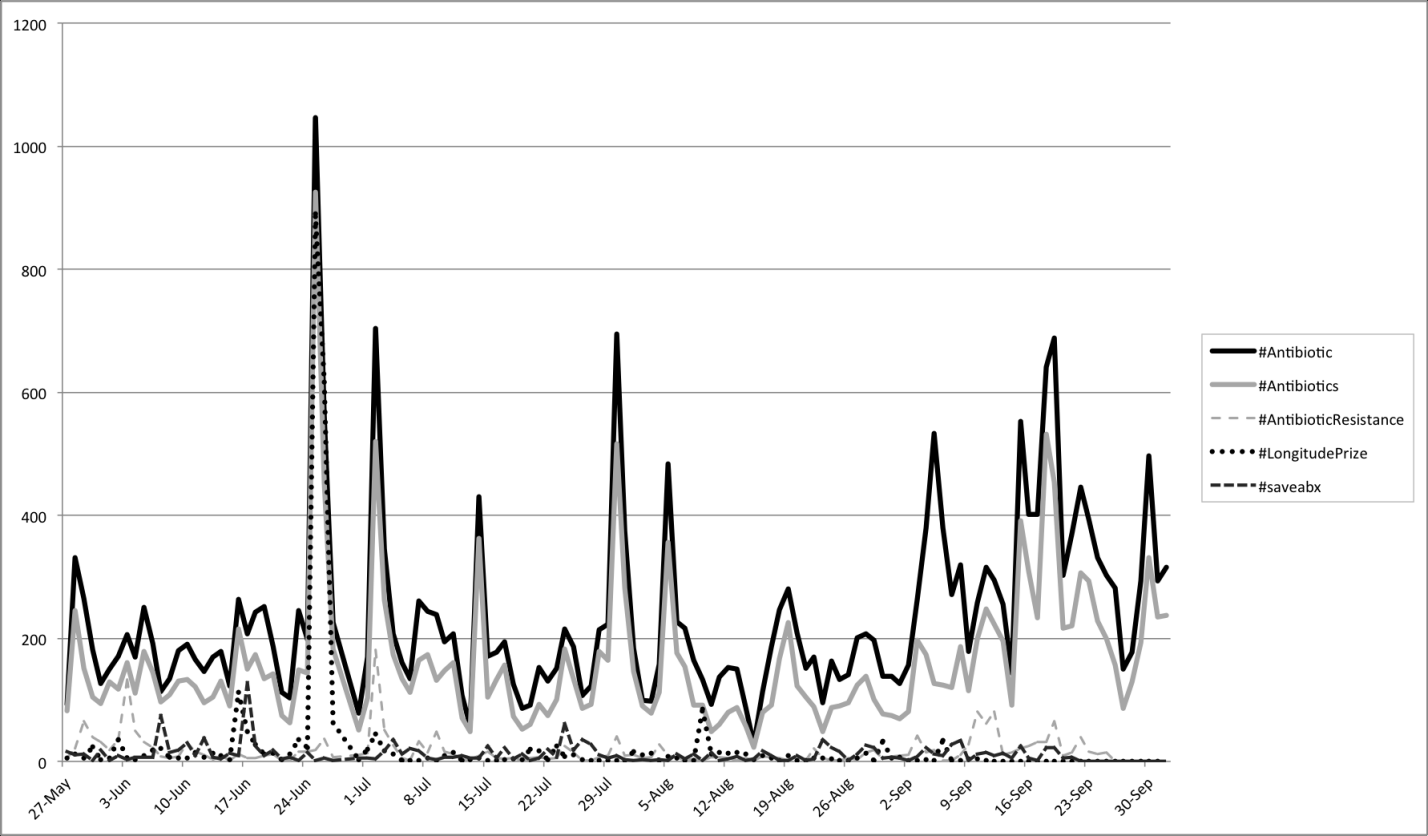
Usage of top overall antibiotic-related hashtags by day.

To further characterize the content of the tweets collected, data on the hashtags used as well as their relative usage and distribution over time was calculated. Table 3 shows the top hashtags by usage. In total, there were roughly 27,458 distinct hashtags used a total of 228,451 times. The vast majority of hashtags (98.94%, 27,166/27,458) were used less than 100 times. Figure 4 depicts the usage of these top hashtags by day over the course of the collection period. As expected, many days that showed spikes in the overall number of antibiotic-related tweets also showed spikes in hashtag usage for #antibiotic or #antibiotics.

**Table 3:** Usage of the most common hashtags from collected set of antibiotic-related tweets.

Rank	Hashtag	Count (Relative frequency)
1	antibiotics	20706 (9.0%)
2	antibiotic	9329 (4.1%)
3	health	7224 (3.2%)
4	longitudeprize	2693 (1.2%)
5	penicillin	2537 (1.1%)
6	antibioticresistance	2168 (0.9%)
7	news	1942 (0.8%)
8	saveabx	1495 (0.7%)

The final query-based analysis that was performed on the entire collection of tweets was with respect to specific messages and users. In particular, the interest was in what, if any, specific messages were being shared (via retweets) and if the overall exchange taking place in the Twittersphere was being dominated by a set of users. The number of retweeted messages was substantial (27.90% of all tweets were retweets [164,973/591,091]), but most retweets had a limited reach (ie, only 11 tweets had more than 500 retweets). Many of the most retweeted messages were advertisements or concerns about mixing antibiotics and agriculture. None of the tweets went “viral”. As for the source of the tweets, the collection included 327,930 different Twitter users. This value was determined through a HiveQL query to collate the tweets by screen name (ie, a unique identifier for a Twitter account) and count the number of tweets per name. Only 0.01% of these users (4255/327,930) contributed 10 or more tweets, yet this small number of users was responsible for 22.68% (134,081/591,091) of all collected tweets.

### Characterizations of Tweets Through Classification

By applying the classifier to the entire dataset and assigning each tweet to the highest scoring category, it is possible to estimate the overall frequency of each category. In particular, it was determined that “Advice/Information” and “Other” were the most predicted categories, accounting for 24.47% (144,627/591,091) and 41.00% (242,318/591,091) of all tweets, respectively. “General Use” and “Resistance” each garnered about 12% (70,253/591,091 for General Use and 72,486/591,091 for resistance) of the overall number of tweets with the remaining categories each receiving less than 5%.

### Evaluation of Classification

The tweet classifier was evaluated on a per-class and all-classes level using the labeled data and the previously described 10-fold cross validation. On the all-class level, a tweet was assigned to the highest scoring category. The percentage of labeled tweets that were correctly classified was 70.4% (293/416). This value is well above what would be expected by chance by a random predictor (eg, ~11% on a balanced dataset) or by a naïve predictor that always predicted the most common class (ie, a classifier that predicted every example as “resistance” would achieve ~30.5%). On a per-class level, the score for each example was used to evaluate the model with respect to a binary classification task. In this setting, the predicted score for a class (eg, “resistance”) was used in conjunction with a threshold to classify the tweet as “resistance”-related or not “resistance”-related. The recall (ie, percentage of tweets pertaining to a class recovered), precision (ie, percentage of tweets correctly predicted to be in a class), and fall-out (ie, percentage of tweets not pertaining to a class that were incorrectly predicted as pertaining to the class) were calculated for each class at varying thresholds and used to calculate the area under the receiver operating characteristic (ROC) curve (AUC). The AUC is used to characterize the effectiveness of a binary classifier regardless of threshold. Table 4 lists the AUC for each class. Note that the AUC of a random predictor would be 0.5. The break-even point (ie, precision=recall) was also calculated for several classes and shown in Table 4. A break-even point of 67, for example, would indicate that it would be expected to recover 67% of the tweets pertaining to the class “Advice/Information” and of those predicted as “Advice/Information”, 67% of the tweets would be correctly classified. Note that due to the small number of labeled examples for some of the categories (eg, “Misuse”, “Side Effects”), it was difficult determine the break-even point.

**Table 4:** The AUC and break-even values for per-class evaluation.

Category	AUC	Break-even point (%)
Advertisement	0.78	~45
Advice/Information	0.87	~67
Animals	0.96	~70
General use	0.95	58
Other	0.89	64
Resistance	0.96	92
Side effects	0.92	-
Wanting/Needing	0.88	29
Misuse	0.80	14

When we applied our classifier to the additional evaluation data of 246 tweets, it correctly classified 68.4% of tweets (76/111) when using a threshold of 0.75 (ie, only those predictions with a score of 0.75 or more for a particular class were considered). When considering all 246 tweets, the accuracy on this set was 48.0% (118/246). This value is lower than the 70% obtained through the 10-fold cross validation. On closer inspection of the tweets contained in the evaluation sample, it was determined that several of the tweets could not be confidently placed in one class. When considering the top 2 predicted classes, the accuracy (ie, percentage of tweets whose top 2 predicted categories corresponded to the manual classification) was 64.2% (158/246).

## Discussion

### Common Source and Content of Collected Tweets

Over the 3-month period during which data were collected, users posted a number of opinions, feelings, and information on many antibiotic-associated topics. Nevertheless, when considering the questions of what particular messages dominated the virtual discussion and who were the primary participants, a few trends are distinguishable. First, a commonly occurring topic during this time period was “resistance”. This is evident by the large number of overall tweets classified as relating to “resistance” (ie, ~10%) and the relative frequency and distribution of the hashtag “#antibioticresistance”. Another trend that shows through is the dominance of several news stories as an origination point for posts. Many of the days with high post counts coincide with national news events related to antibiotics. These tweets often contained a short lead-in and then a URL, indicating that they were generated by embedded Web-links aimed to help users share content through Twitter. This activity, as well as the fact that only a small number of tweets were retweeted more than 500 times and that more than 75% of all tweets collected came from users who generated fewer than 10 tweets from those collected, indicates that this discussion is not being driven by a few individuals but is more organic in nature. It is also clear that specific topics (eg, an announcement from a national leader) have a relatively short duration and with each spike in activity, the amplified number of posts greatly decreases just as quickly as it increased; most spikes lasted only one day.

### Efficient Mining of Tweets

Given the performance of the constructed classifier (ie, 70% percent of the multi-class predictions were correct and reasonable values for the AUC values for the binary classification tasks), it is possible to effectively access larger amounts of tweets in a manageable fashion. In particular, it is possible to more efficiently peruse tweets by category by varying the decision threshold. Taking advantage of the classic trade-off between precision and recall, one can retrieve a small number of confident predictions or sift through a larger number of less confident predictions. Table 5 contains a sample of tweets, their predicted category, and score.

**Table 5:** Sample tweets that were discovered through the trained classifier: tweet text, predicted category, and score.

Category (score)	Tweet text
Need (0.85)	I’ve never been so excited to go to the doctor to get antibiotics
Need (0.83)	I hate doctors so much, I shouldn’t have to demand antibiotics ffs
Need (0.70)	Any of my local friends have antibiotics laying around they haven’t finished? Don’t have ins & can’t afford to go to the docr
Misuse (0.85)	Ive had stepthroat for a month and I will throw the biggest …… fit this doctors office has ever seen if they don’t give me antibiotics
Misuse (0.77)	I’m on antibiotics for this sinus infection which means no drinking. Guess what I’m doing?
Side Effects (0.70)	Common antibiotic may increase heart death risk URL
Side Effects (0.70)	I’ll never take an antibiotic before I go to sleep again, my body was itching all ……. night?
Side Effects (0.85)	@.... I am now. We found out that the antibiotic I was on causes sever motion sickness. Lol. I just can’t drive while I’m taking them.
Side Effects (0.95)	So sick I think I’ve turned green, never take antibiotics on an empty stomach. Learned my lesson. ??
Advice/Information (0.70)	Bacteria can evolve a biological timer to survive antibiotic treatments – Medical News Today URL
Advice/Information (0.80)	UK says …… recalls batches of Indian-made antibiotic ????
Advice/Information (0.91)	U.S. Congress urged to pass bill to speed development of antibiotics #Health care

To further investigate the use of the classifier, it was used to identify and visually inspect tweets classified as pertaining to “Misuse”. Tweets classified as pertaining to “Misuse” were collected using thresholds of 0.3, 0.4, 0.5, 0.6, 0.7, and 0.8 which yielded 2623, 1152, 510, 205, 42, and 8 tweets respectively. Given the relatively short nature of the tweets, all tweets with a score of over 0.3 were read. In examining these tweets, a number of user messages mentioned mixing alcohol and antibiotics, missing and/or recuperating missed doses, taking antibiotics without a meal, and taking and/or looking for antibiotics for influenza.

### Comparison With Existing Tools and Approaches for Mining the Twittersphere

Given the value of user-generated content, a number of enterprises have developed proprietary tools to collect, process, and provide access to Twitter data (eg, Topsy and Talkwalker). Access is typically provided through a Web interface or API and may come at a cost. A comprehensive comparison between these commercial options and the methods developed in this work is difficult due to limited access and the speed at which these tools evolve to meet market needs. Nevertheless, there are clear advantages and disadvantages to each approach that can be discussed in general terms. Commercial tools often provide access to historical Twitter data and can be used to perform retrospective studies. The Web interfaces provided make some tweets and data derived from the tweets easy to access and visualize in a manner that does not involve extensive technical knowledge (eg, to manage or query the data). The drawbacks to these tools include the monetary cost of gaining access to the tool and/or data and restricted access to the data. The open-source solution described here does provide finer-grain access to the data since all data pertaining to a tweet are stored locally. There is also no cost associated with software tools used as they are open source and liberally licensed. The principle drawback is the added technical knowledge needed to manage the data and use the tools.

Apart from the cost, the level of access to the data is perhaps the most important distinction between the two approaches. As an example, consider some of the data mining and analysis tasks performed in this study. These included counting the number of relevant tweets per day, determining the source of spikes in related traffic, and determining common contributors to the virtual discussion. These tasks could have readily been accomplished with commercial tools. By having direct access to the data and software to perform queries, it was possible to also determine what specific words and word pairings were also common in tweets relating to antibiotics. This information was used to build the classifier and also identify topics of discussion on days of peak activity. It was also possible to drill down on hashtags and hashtag usage among tweets about antibiotics. Thus, more control and more complex queries could be executed.

With regards to findings, this work confirms and complements existing approaches for mining content related to antibiotics from the Twittersphere. In a study of Twitter and antibiotics, Scanfeld et al derived categories and manually classified 1000 tweets [11]. The relative frequencies of tweets from several categories (eg, misuse, needing/wanting) were similar to those obtained in this study with the exception of resistance, which had increased since 2010. Furthermore, Scanfeld et al used several keyword pairs (eg, “extra” and “antibiotics”) to search for specific instances or details of misuse. Using the tools and approaches presented in this work, it is possible to extend the discovery process by identifying common keyword pairs (ie, query to determine what words commonly co-occur, possibly identifying new types of misuse) or searching through tweets by predicted category (ie, the tweet may lack the suspected keywords associated with misuse but be predicted as pertaining to misuse through the learned patterns). Dyar et al used a commercial tool (ie, Topsy) to study spikes in Twitter activity surrounding tweets that mentioned “antibiotics” [9]. These spikes were found to be short-lived and driven by media coverage of governmental action. This phenomenon was also seen in the aggregate collection of tweets (ie, not only those with the keyword “antibiotic” but all tweets collected for this study) and also present in hashtag usage.

### Limitations

This study, and indeed analyses of large amounts of Twitter data, is not without its limitations. First, the data are very noisy. In this study for example, additional post filtering was needed to remove tweets that were related to the stock ticker “ABX”. Collecting tweets by keywords is difficult as miscellaneous or tangential data can easily be selected (eg, a joke mentioning antibiotics). These extraneous tweets can easily skew the dataset if they become popular and are retweeted several times. Additionally, the tweets are by their nature quite short and full of abbreviations, and this can make their interpretation ambiguous and difficult to interpret without the benefit of additional text to give context. In some cases, qualitative judgments had to be made as to which category a tweet belongs since the categories chosen were not mutually exclusive. This was a limitation of the machine learning approach we employed. Future works could break all needed categorizations into mutually exclusive sets and train a classifier for each set.

Another limiting factor to this study and approach is its specialization. While the approach is general enough to be applied to a number of topics and domains in the Twittersphere, a new model would need to be trained and a new feature set selected. This is because the data collected would have to be analyzed to determine what words would be useful for the bag-of-words characterization.

### Conclusions

This study developed and implemented means to characterize the larger discussion taking place on Twitter with respect to a particular health-related topic (eg, antibiotics). Using tools such as Hive, Flume, Hadoop, and machine learning techniques, it is possible to collect and query large amounts of Twitter data to determine what words, phrases, or contributors were dominating the online discussion. It is also possible to identify and characterize of periods of high activity. In this study in particular, it was determined that several national actions with respect to antibiotics led to several spikes in activity. Furthermore, using newer machine learning techniques and a limited number of manually labeled tweets, the entire body of collected tweets can be classified to indicate what topics are driving the discussion. The classifier can also be used to efficiently explore collected tweets by category and search for messages of interest or exemplary content.

## Abbreviations

API: application programming interface
AUC: area under the ROC curve
ROC: receiver operating characteristic
SQL: structured query language
URL: uniform resource locator
